# Overexpression of *CsSAMT* in *Citrus sinensis* Induces Defense Response and Increases Resistance to *Xanthomonas citri* subsp. *citri*

**DOI:** 10.3389/fpls.2022.836582

**Published:** 2022-03-24

**Authors:** Cesar Augusto Nascimento, Natalia Sousa Teixeira-Silva, Raquel Caserta, Marcia Ortiz Mayo Marques, Marco Aurelio Takita, Alessandra A. de Souza

**Affiliations:** ^1^Citrus Research Center “Sylvio Moreira”, Agronomic Institute – IAC, Cordeirópolis, Brazil; ^2^Department of Genetics, Evolution and Bioagents, Institute of Biology, University of Campinas – UNICAMP, Campinas, Brazil; ^3^Department of Phytochemistry, Agronomic Institute – IAC, Campinas, Brazil

**Keywords:** systemic-acquired resistance, salicylic acid, methyl salicylate, MeSA, citrus canker

## Abstract

Citrus canker is a destructive disease caused by *Xanthomonas citri* subsp. *citri*, which affects all commercial sweet orange (*Citrus sinensis* [L.] Osbeck) cultivars. Salicylic acid (SA) and systemic-acquired resistance (SAR) have been demonstrated to have a crucial role in mediating plant defense responses against this phytopathogen. To induce SAR, SA is converted to methyl salicylate (MeSA) by an SA-dependent methyltransferase (SAMT) and translocated systemically to prime noninfected distal tissues. Here, we generated sweet orange transgenic plants (based on cvs. Hamlin and Valencia) overexpressing the *SAMT* gene from *Citrus* (*CsSAMT*) and evaluated their resistance to citrus canker. We obtained four independent transgenic lines and confirmed their significantly higher MeSA volatilization compared to wild-type controls. Plants overexpressing *CsSAMT* showed reduced symptoms of citrus canker and bacterial populations in all transgenic lines without compromising plant development. One representative transgenic line (V44SAMT) was used to evaluate resistance response in primary and secondary sites. Without inoculation, V44SAMT modulated *CsSAMT*, *CsNPR1*, *CsNPR3*, and *CsWRKY22* expression, indicating that this plant is in a primed defense status. The results demonstrate that MeSA signaling prompts the plant to respond more efficiently to pathogen attacks and induces immune responses in transgenic plants at both primary and secondary infection sites.

## Introduction

Citrus canker is a severe bacterial disease caused by *Xanthomonas citri* subsp. *citri*, which affects all commercial citrus cultivars and causes extensive economic damage worldwide (Gochez et al., 2020; Martins et al., 2020; Behlau, 2021). Most commercial sweet orange cultivars are moderately to highly susceptible to citrus canker (de Carvalho et al., 2015; Mei et al., 2020). Currently, copper-based chemicals are the main compounds used to control citrus canker; however, their excessive use leads to environmental issues, plant stress, and bacterial resistance (Lamichhane et al., 2018; Behlau et al., 2020; Heydarpanah et al., 2020). To overcome these problems, biotechnology-based approaches have shown potential to increase plant resistance against citrus canker in a sustainable way (Caserta et al., 2020; Salonia et al., 2020; Soares et al., 2020).

Salicylic acid (SA) is a plant hormone that plays a crucial role in both local immune responses and systemic-acquired resistance (SAR) (Li et al., 2019; Peng et al., 2021). In plants, SA is synthesized *via* two distinct pathways: through the isochorismate synthase (ICS) pathway in plastids and the phenylalanine ammonia-lyase (PAL) pathway in the cytosol (Wildermuth et al., 2001; Cochrane et al., 2004; Strawn et al., 2007; Zhang and Li, 2019; Lefevere et al., 2020). SA is essential to protect plants against biotrophic and hemibiotrophic pathogens, such as *X. citri* (Wang and Liu, 2012; Le Thanh et al., 2017; Islam et al., 2021; Peng et al., 2021). Citrus plants pretreated with exogenous SA showed significantly enhanced endogenous SA levels that led to a reduction in the *X. citri* incidence rate (Wang and Liu, 2012). Similarly, soil applications of the SA analog acibenzolar-S-methyl (ASM), a known SAR-inducing compound, have also contributed to the control of citrus canker disease (Graham and Myers, 2011; de Mello et al., 2020).

SAR is a broad-spectrum resistance of plants induced in distal tissues by the signal compounds produced in the primary infected leaves (Wenig et al., 2019; Gao et al., 2021). *NPR1 (Non-expressor of pathogenesis-related genes 1)* is a key gene required for SA defense responses and SAR activation (Backer et al., 2019). The overexpression of *NPR1* from *Arabidopsis thaliana* (*AtNPR1*) and its homolog from *Citrus maxima* (*CtNH1*) increased resistance to citrus canker in Duncan grapefruit (*Citrus paradisi* Macfadyen), Hamlin sweet orange (*C. sinensis*), and pummelo (*Citrus maxima*), respectively (Zhang et al., 2010; Chen et al., 2013; Boscariol-Camargo et al., 2016). *NPR1* responds to increased levels of SA, by controlling the expression of a set of SA-regulated genes, which triggers the synthesis of defense-related proteins such as pathogen-related (PR) proteins (Kumar, 2014) and WRKY transcription factors (Phukan et al., 2016; Chen et al., 2019). However, NPR3 and NPR4 exert an antagonistic action on NPR1, by inducing its degradation *via* the proteasome, thus acting as SA negative regulators (Castelló et al., 2018; Ding et al., 2018).

A few mobile secondary molecules have been reported as elicitors of SAR, including methyl salicylate (MeSA) (Fu and Dong, 2013; Gao et al., 2021). MeSA is a mobile signal compound derivative of SA synthesized by SA-dependent methyltransferase (SAMT) (Zubieta et al., 2003). MeSA is translocated from the primary infection site *via* the phloem to reach distal tissues and trigger systemic immunity responses (Park et al., 2007; Gao et al., 2021). In distal leaves, MeSA is reconverted into SA by methyl esterase SA-Binding Protein 2 (SABP2), activating SA-related defense genes to induce SAR (Forouhar et al., 2005; De Lima Silva et al., 2019; Ding and Ding, 2020). Tomato plants overexpressing *SlSAMT* accumulated higher levels of SA and MeSA and exhibited slightly delayed symptom development upon *Xanthomonas campestris* pv. *vesicatoria* infection (Tieman et al., 2010). Similarly, the overexpression of *GmSAMT1* in soybean conferred broad resistance to multiple soybean cyst nematode races (Lin et al., 2013, 2016). We previously demonstrated that the overexpression of the *SAMT* gene from *Citrus reticulata (CrSAMT)* in tobacco *(Nicotiana tabacum)* showed a significant symptom reduction in response to *Xylella fastidiosa* subsp. *pauca* (Gómez et al., 2020). In addition, *CrSAMT*-transgenic plants increased MeSA volatilization, promoting the induction of PR1 in distal tissues and in neighboring wild-type plants (Gómez et al., 2020). Recently, overexpression of *CsSAMT1* (homologous to *CrSAMT*) in Wanjincheng oranges (*C. sinensis*) was demonstrated to enhance the tolerance to Huanglongbing caused by *Candidatus* Liberibacter asiaticus (LAS) in citrus species (Zou et al., 2021).

We previously showed that *C. reticulata* CrSAMT belongs to the SABATH family of methyltransferases containing SAM- and SA-binding residues (Gómez et al., 2020). An identical copy of CrSAMT is present in *C. sinensis* (*CsSAMT*) [Cs1g24440.1 v2.0 (HZAU), orange1.1g017514m v1.1 (JGI) and XM_006466773 (NCBI)] (Gómez et al., 2020). Thus, considering previous reports regarding the role of SAMT in the defense response against citrus bacterial pathogens (Gómez et al., 2020; Zou et al., 2021), we generated stable Valencia and Hamlin sweet orange transgenic lines overexpressing *CsSAMT* and evaluated local and systemic resistance against *X. citri*. In addition, we explored the potential of MeSA to modulate defense-related genes.

## Materials and Methods

### *CsSAMT* Vector and *C. sinensis* Genetic Transformation

The binary vector pCambia2201 containing the target gene *CrSAMT* from *C. reticulata* was produced as described by Gómez et al. (2020). Because the *CrSAMT* sequence is identical to *CsSAMT* used in other studies (Martini et al., 2018; Zou et al., 2021), we will use *CsSAMT* to standardize and avoid misunderstanding concerning gene function. The *Agrobacterium tumefaciens* EHA105 strain carrying *CsSAMT*_Cambia2201 was used to transform two *C. sinensis* cultivars (Hamlin and Valencia) as previously described (Caserta et al., 2014). Shoots were regenerated in MS basal medium (Murashige and Skoog, 1962) supplemented with 30 g/L sucrose and Phytagel (Sigma-Aldrich, Saint Louis, MO, United States) at 2.5 g/L with 100 μg/ml kanamycin. The regenerated shoots were tested for β-glucuronidase (GUS) activity (Jefferson et al., 1987) when leaves were immersed in 2 mm “X-gluc” solution (5-bromo-4-chloro-3-indolyl-β-d-glucuronic acid) and incubated overnight at 37°C. Destaining was carried out in ethanol and acetic acid (1:1 v/v) to remove chlorophyll and other pigments. Blue tissues were considered positive for transformation. Well-developed shoots were directly grafted into 6-month-old Rangpur lime rootstock (*Citrus x limonia* Osbeck) and transferred to the greenhouse at an average temperature of 28°C.

### Analysis of Transformed Plants

Before transfer to the greenhouse, GUS-positive plants were analyzed by polymerase chain reaction (PCR) using a specific primer pair designed to anneal to the FMV promoter and the CaMV35S terminator to avoid amplification of the endogenous *CsSAMT* gene (Table 1). Genomic DNA was extracted from GUS-positive and WT plants using the hexadecyltrimethylammonium bromide (CTAB) extraction method (Doyle, 1990). The integrity and quality of DNA were verified in an ND-8000 NanoDrop spectrophotometer (Thermo Fisher Scientific, San Jose, CA, United States). PCRs were performed in GoTaq buffer in a final volume of 25 μl containing 100 ng of total genomic DNA, 160 μm dNTPs, 0.2 μm of each primer, and 1.25 U of GoTaq DNA polymerase (Promega, Madison, WI, United States). The PCR steps were 95°C for 5 min; 35 cycles of 30 s at 94°C, 30 s at 58°C, and 60 s at 72°C; and a final extension at 72°C for 10 min. The expected PCR amplicon was visualized in a 1% agarose gel.

**Table 1:** List of primers used for PCR and RT-qPCR detection.

Gene	Description/name	Amplicon (pb)	Primer sequence 5′ - 3′	Accession
*CsSAMT* FMV/TER	*Salicylate carboxy methyltransferase*	1,540	F - GTGGGGACCAGACAAAAAAGGAATG	XM_006466773
			R - CGAAACCCTATAAGACCCTAATTCCC	
*CsSAMT* RTqPCR	*Salicylate carboxy methyltransferase*	100	F - GGAGGTGGTTCAAGTGCTTC	XM_006466773
			R - TGGCTTTGCAATGGATATGA	
*CsNPR1*	*Non-expressor of Pathogenesis-Related gene1*	150	F - GTTGGGTTTGATCCGCTTGT	XM_006475416
			R - AAGAACCTCCACCATAAAATCAACA	
*CsNPR3*	*Non-expressor of Pathogenesis-Related gene3*	150	F - TGAGGGAAAATCAGTGGCTGTT	XM_006468378
			R - CCATAAGGCACCAATTCAGTCA	
*CsICS*	*Isochorismate synthase*	150	F - TGCCATTTTCGGGTTGAAAT	XM_006476588
			R - TTCTTTTCTGGTCGAAGCCG	
*CsPAL1*	*Phenylalanine ammonia-lyase*	125	F - GATGCTCAGGAAGCCTCTAAAC	XM_006481430
			R - GCGTCGAACAGAACCATAGAA	
*CsWRKY22*	*WRKY22 transcription factor*	58	F - GCGGATTGTCTCGCATGTG	XM_006476427
			R - TTATGGGTTTCTGCCCGTATTT	
*CsPR1*	*Pathogenesis-Related Protein 1*	61	F - AAGGAAAGCGGATTGCAAACT	XM_006474081
			R - CTCGCCAAGCTTGAAATTGTC	
*CsUBI*	*Ubiquitin*	100	F - TTCGTCAGTTGACTAATCCT	XM_025095259
			R - GTTGCTGTGTTGACTGTG	

### Gene Expression Analysis Using Quantitative Reverse-Transcriptase PCR

Plants with amplicons of the expected size were subjected to *CsSAMT* gene expression analysis by qRT-PCR. Total RNA was extracted using the PureLink^™^ RNA Mini Kit (Invitrogen, Waltham, MA, United States) according to the manufacturer’s instructions. The RNA quality and concentration were determined by spectrophotometry NanoDrop ND-8000 (Thermo Fisher Scientific, San Jose, CA, United States) and electrophoresis. The absence of cross-contamination with genomic DNA was verified by conventional PCR using RNA as template and endogenous primers (Table 1). One microgram of total RNA was used for cDNA synthesis with a Reverse Transcription GoScript Kit^™^ (Promega, Madison, WI, United States) following the manufacturer’s instructions. Specific primers for qRT-PCR were designed using Primer Express^®^ Software v3.0.1 (Thermo Fisher Scientific, San Jose, CA, United States). The reactions were performed using 6.5 μl of GoTaq^®^ qPCR Master Mix (Promega, Madison, WI, United States), 120 nm of each gene-specific primer pair (Table 1), and 3 μl of 1:24 diluted cDNA in a final volume of 12.5 μl. Amplifications were performed in the QuantStudio 5 system (Applied Biosystems, Foster City, CA, United States) using the following thermal profile: 95°C for 2 min followed by 40 cycles of 95°C for 3 s and 60°C for 30 s. Dissociation curves were analyzed in every amplification to confirm the primer specificity. The primer efficiencies were estimated using LinRegPCR software (Ramakers et al., 2003), and relative gene expression was determined by the 2^ΔΔCt^ method (Livak and Schmittgen, 2001). The *ubiquitin* (*UBI*) gene was used as an internal control for transcript normalization (Mafra et al., 2012). Experiments were performed in triplicate, and the statistical significance of the means was calculated according to Student’s *t*-test (^*^p < 0.05) in GraphPad Prism 9 for Windows (GraphPad Software, San Diego, CA, United States).

### MeSA Extraction and Quantification

The volatilized MeSA was detected and quantified using the dynamic headspace method (Gómez et al., 2020) with modifications. Three biological replicates from both 6-month-old *CsSAMT* lines and WT plants were placed into glass flasks arranged in parallel directly connected to a glass tube packed with Super-Q absorbent (100 mg, HayeSep Q 80/100—Ohio Valley Specialty). Leaf volatiles were continuously captured under vacuum (640 mm Hg) for 24 h (Supplementary Figure 1). The volatiles trapped in the Super-Q absorbent were eluted in 400 μl of dichloromethane. For each sample, 1 μl was manually injected in splitless mode into a gas chromatograph/mass spectrometer (GC/MS, Shimadzu, QP-5000). The GC was equipped with a fused silica DB-5 capillary column (J&Wiley Scientific, 30 m × 0.25 mm × 0.25 mm), with an injector temperature of 220°C and helium as the carrier gas at a constant flow of 1.0 ml/min. The oven temperature conditions started at 35°C for 2 min, increased to 170°C at a rate of 3°C/min, then increased to a final temperature of 280°C at a rate of 15°C/min, and held for 2 min. The mass spectrometer conditions were as follows: interface temperature at 240°C, ionization voltage of 70 eV, mass range 41–250 *m/z*, and selected ion monitoring (SIM) mode (selected ions: *m/z* 92, *m/z* 120, and *m/z* 152). The identification of MeSA in plants was performed by comparison with the mass spectra of the GC/MS system database (NIST 62 Library) and commercial standard (Sigma-Aldrich, ≥ 99%, chromatographic grade, Lot MKBP7145V). In addition, the retention times of MeSA were compared with the retention times of the methyl salicylate standard. The quantification of MeSA in the samples was carried out by the external standard method in GC/MS (mode SIM, selected ions: *m/z* 92, *m/z* 120, *m/z* 152). This procedure was performed based on the peak area of the ion at *m/z* 120, which was used as the target ion, followed by the ions at *m/*z 92 and *m/z* 152 as reference ions to confirm the compound specificity. The calibration curve was prepared with a commercial methyl salicylate standard diluted in dichloromethane (chromatographic grade) at the following concentrations: 1.2 μg/ml; 1.0 μg/ml; 0.8 μg/ml; 0.6 μg/ml; 0.4 μg/ml; and 0.2 μg/ml. Aliquots of 1 μl of each solution were injected and analyzed under the same chromatographic conditions as the samples in triplicate.

The concentration of MeSA was calculated by measuring the areas of each MeSA peak and plotting in a standard curve (Supplementary Figure 2), according to Engelberth et al. (2003). Experiments were performed in triplicate, and the statistical significance of the means was calculated according to Student’s *t*-test (*p* < 0.05 ^**^ < 0.01).

### Evaluation of Citrus Canker Symptoms in Transgenic Lines

Three fully expanded leaves of similar age and size per line were infiltrated with *X. citri* strain 306 carrying a GFP marker gene (Rigano et al., 2007). *X. citri* was grown overnight in liquid NBY nutrient medium (0.5% peptone, 0.3% meat extract, 0.2% yeast extract, 0.2% K_2_HPO_4_, and 0.05% KH_2_PO_4_) containing ampicillin at 100 μg/ml and gentamicin at 5 μg/ml. Three regions on each leaf surface were pierced with a needle (inoculation point) and infiltrated with an *X. citri* suspension (10^4^ CFU/ml) in phosphate saline buffer (PBS) using a needleless syringe. Symptoms and bacterial populations (CFU/mL) were evaluated at 7 and 14 days after inoculation (DAI). Phenotypic analysis of the canker lesions was performed using an Olympus MVX10 stereomicroscope with a U-MGFPHQ filter to detect GFP. Leaf disks were excised exactly at the inoculation point and out of the inoculation point within each inoculated region (Supplementary Figure 3). Disks were homogenized in 1 ml of PBS, serially diluted and plated on selective NBY agar plates for bacterial population analysis. At least three independent biological experiments with four internal replicates were performed, and the statistical significance of the means was analyzed using Student’s *t*-test (*p* < 0.05) in GraphPad Prism 9 for Windows (GraphPad Software, San Diego, CA, United States).

### Systemic Resistance Assay

**Figure 1:**
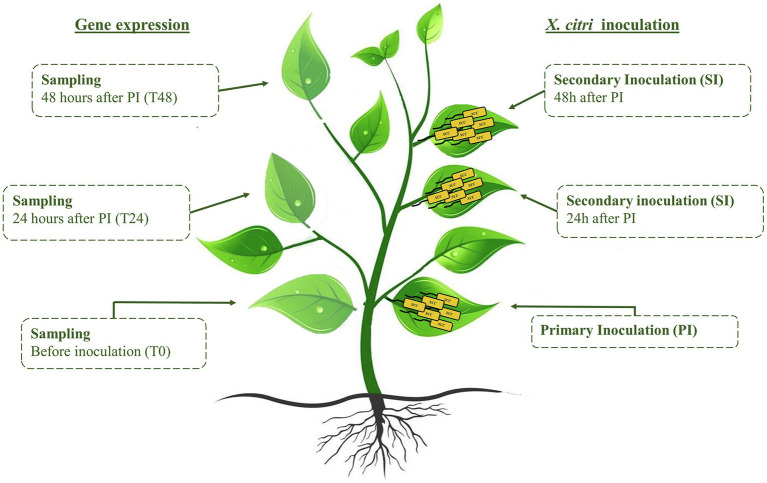
Representative scheme of systemic activation assay. Sampling for gene expression analyses was carried out immediately before inoculation of *Xanthomonas citri*. Therefore, sampling at T0 was followed by primary inoculation; sampling at T24 and T48 preceded the 24 h and 48 h secondary inoculations, respectively.

To induce systemic responses in *C. sinensis* plants, primary inoculation of *X. citri* was carried out on a leaf surface (0 h), and then, after 24 and 48 h, secondary inoculations were performed on distal leaves (Figure 1). The inoculations were performed using a developed pin-roller method as described. A pin-roller (Dermaroller 540 System—ELON-YC, China) containing 540 microneedles 0.5 mm in length was rolled over the leaf abaxial surface. These microinjuries allowed bacterial entrance while minimizing mechanical damage, reducing experimental variation (Supplementary Figure 4). The leaves were moistened with sterile absorbent cotton wool presoaked in a 10^8^ CFU/ml bacterial suspension or in PBS as a negative control. The inoculated plants were placed in humidity chambers and kept at 28°C. Symptoms and bacterial populations were evaluated 7 days after the primary and secondary inoculations. Canker pustules were photographed under bright field using an Olympus MVX10 (U-MGFPHQ filter) stereomicroscope. Four areas were analyzed for canker lesions by measuring the pixels using ImageJ software (Schneider et al., 2012; Supplementary Figure 4). At least three biological experiments with four internal replicates were performed using Student’s *t*-test (*p* < 0.05) in GraphPad Prism 9 for Windows (GraphPad Software, San Diego, CA, United States). In addition, noninfected leaves were evaluated from a representative transgenic plant and the wild type for expression analysis of defense-related genes (Table 1). The samples were collected immediately before the primary and secondary stages (24 and 48 h) and named T0, T24, and T48, respectively (Figure 1). The leaves were immediately frozen in liquid nitrogen and stored at −80°C for RNA extraction as described above.

## Results

### Successful Integration of *CsSAMT* in the Sweet Orange Genome

**Figure 2:**
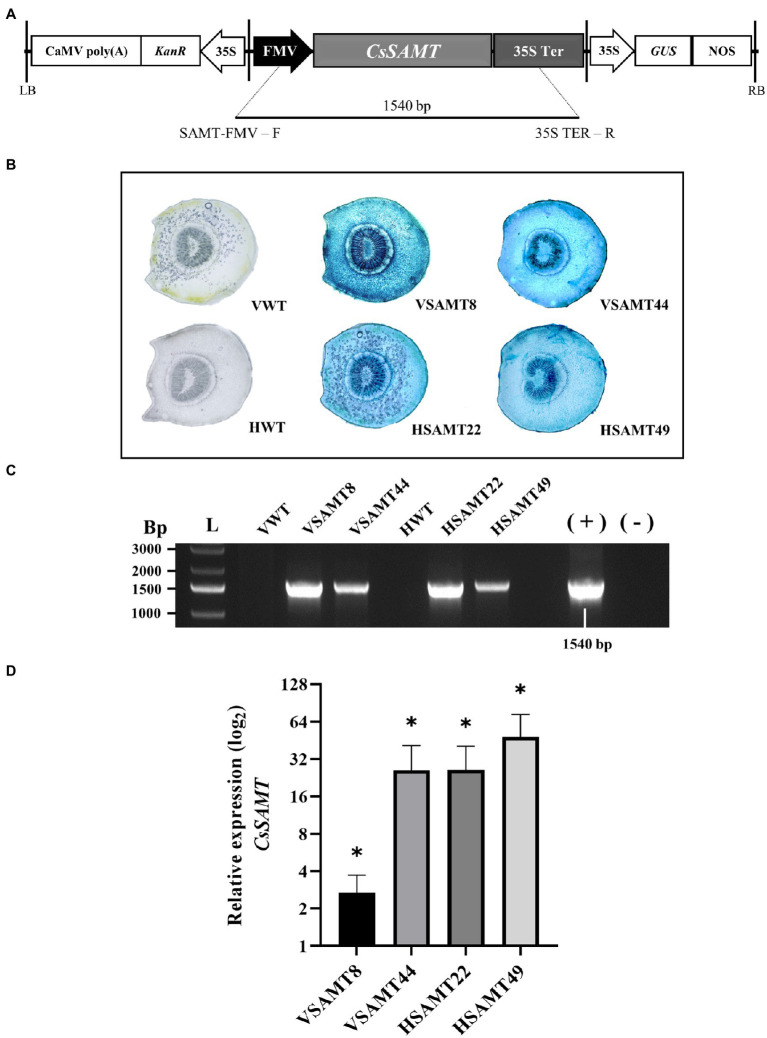
Identification of transgenic sweet orange plants overexpressing *CsSAMT*. **(A)** T-DNA region of the binary vector used for *Agrobacterium tumefaciens*-mediated transformation; **(B)** GUS assay of transgenic *CsSAMT* petioles, wild-type Valencia (VWT), and Hamlin (HWT) as negative controls. **(C)** Amplification of a 1,540 bp fragment of the transformed lines; (L) - GeneRuler 1 kb Plus DNA Ladder (Sigma–Aldrich); Valencia (VWT) and Hamlin (HWT) nontransformed controls, respectively; transformants from Valencia (VSAMT8 and VSAMT44); − transformants from Hamlin (HSAMT22 and HSAMT49); (+) – positive control; (−) – negative control; and **(D)** Relative expression of *CsSAMT* in transgenic sweet orange with nontransformed plants used as control. Three biological independent experiments were performed. Asterisks (^*^) indicate statistically significant differences based on Student’s *t*-test (*p* < 0.05).

The *CsSAMT* gene from *Citrus* was successfully introduced into Valencia and Hamlin sweet orange cultivars (*C. sinensis* L. Osb.) by *Agrobacterium tumefaciens*-mediated transformation using the *CsSAMT*_Cambia2201 vector (Gómez et al., 2020; Figure 2A). Two transformants from Valencia (VSAMT8 and VSAMT44) and two from Hamlin (HSAMT22 and HSAMT49) cultivars were obtained and confirmed by histochemical assay (GUS) (Figure 2B) and PCR (Figure 2C). The expression of *CsSAMT* was determined by qRT-PCR, and significantly increased gene expression was observed in all four sweet orange transgenic lines (Figure 2D). Ten buds from each of these plants were used for grafting into Rangpur lime plants for subsequent studies. To identify the presence of chimeras, β-glucuronidase (GUS) assays were performed on the leaves of propagated plants as previously described (Caserta et al., 2014). Only GUS-positive nonchimeric plants were used in the experiments. The regenerated plants exhibited normal phenotypes with no obvious abnormalities (Supplementary Figure 5).

### *CsSAMT* Overexpression Increases MeSA Volatilization in *C. sinensis*

**Figure 3:**
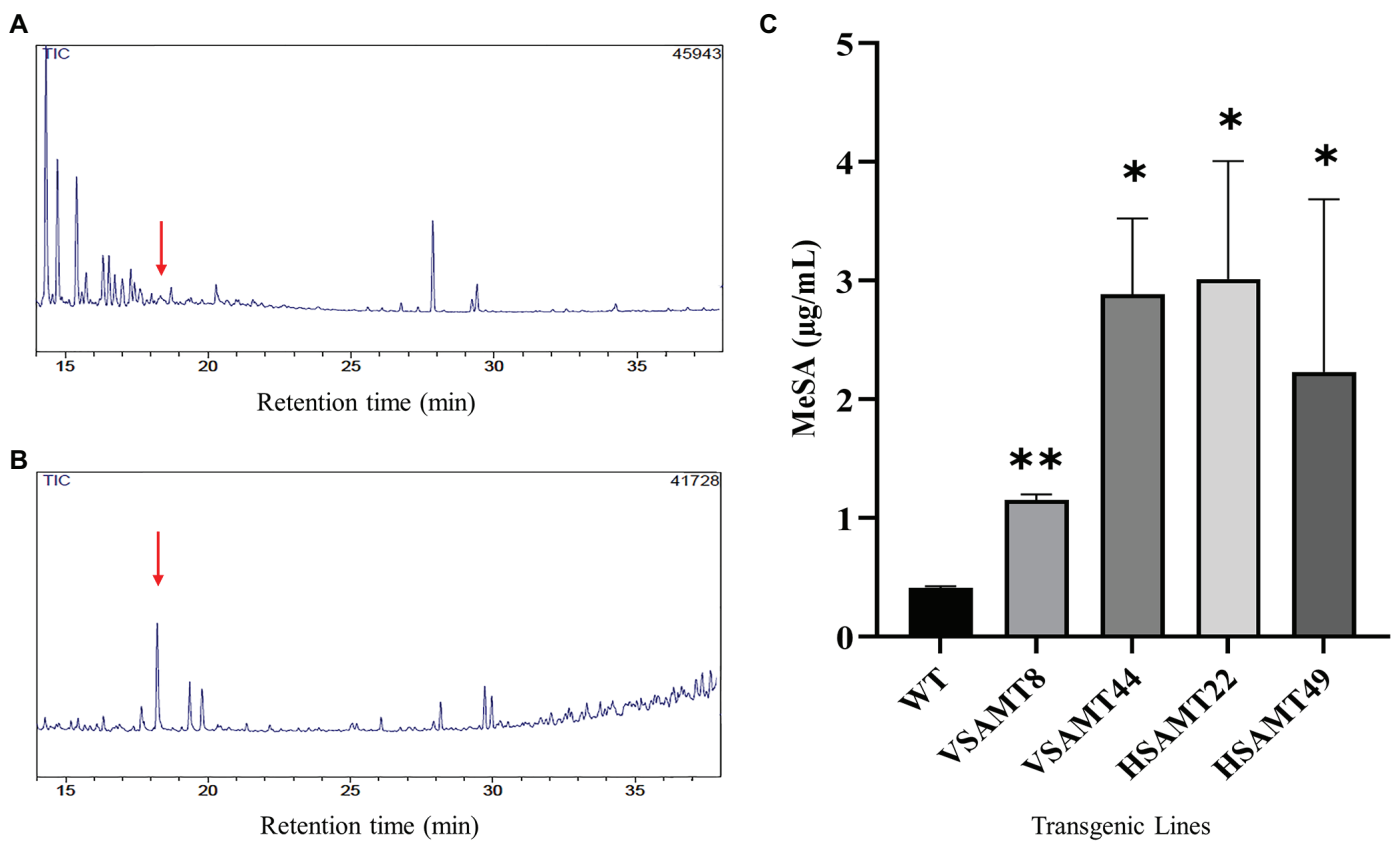
MeSA production in transgenic plants. Representative chromatograms acquired from WT **(A)** and transgenic plants overexpressing *CsSAMT* (HSAMT22; **B)** in GC/MS in SIM mode. Red arrows represent the MeSA peak at a retention time of 18.3 min. **(C)** Determination of methyl salicylate (MeSA) production in the CsSAMT lines and WT plants. Bars represent the means of three biological replicates extracted from entire plants using a headspace method and analyzed by GC/MS. Asterisks indicate statistically significant differences based on Student’s *t*-test (^*^p < 0.05; ^**^p < 0.01) in relation to the values obtained for WT.

To assess MeSA production and volatilization in the transgenic lines, the plants were evaluated by GC/MS. The MeSA retention time in the GC/MS was established at 18.304 min using the commercial standard and selected ion monitoring (SIM) technique (Supplementary Figure 6), which enabled MeSA detection even at low concentrations. For quantitation, a standard curve was generated by using dilutions of known amounts of standard MeSA diluted in dichloromethane at ranges varying from 1.2 μg/ml to 0.2 μg/ml with an R^2^ of 0.9912 (Supplementary Figure 2). The analysis confirmed higher MeSA production and volatilization in transgenic plants overexpressing *CsSAMT* than in the WT (Figures 3A,B). The increase in volatilized MeSA was significant for all transgenic plants overexpressing *CsSAMT*, with WT plants showing a MeSA concentration of 0.41 μg/ml compared to concentrations of 1.15 μg/ml in VSAMT8; 2.85 μg/ml in VSAMT44; 3.01 μg/ml in HSAMT22; and 2.23 μg/ml in HSAMT49 (Figure 3C). These results confirmed that overexpressing *CsSAMT* increases MeSA production and volatilization, in accordance with what was previously reported (Gómez et al., 2020; Zou et al., 2021).

### Overexpression of *CsSAMT* Decreases Canker Symptoms

**Figure 4:**
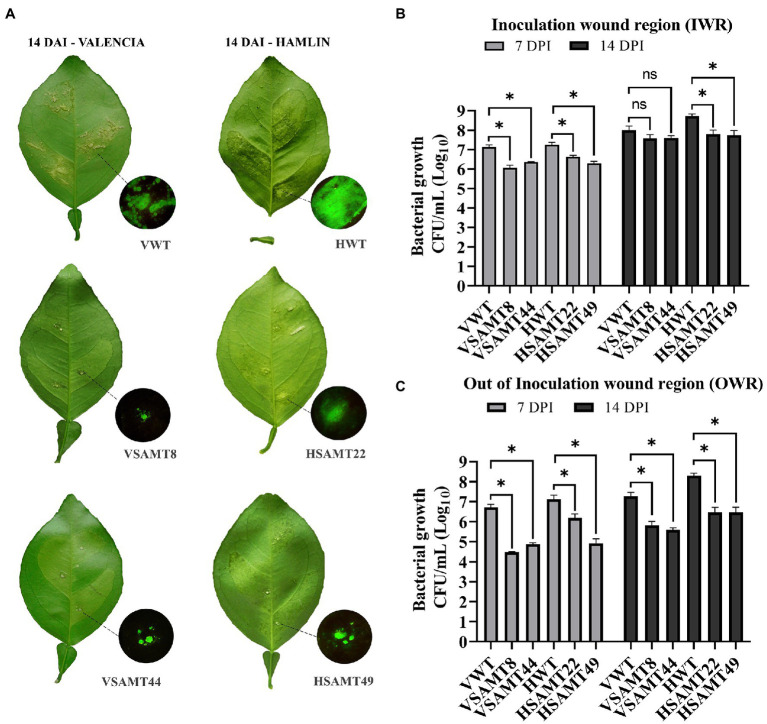
Symptoms and bacterial population in transgenic *CsSAMT* lines and WT. **(A)** Fully expanded leaves of *CsSAMT* lines and the wild type (VWT and HWT) infiltrated with *X. citri* 306_GFP (10^4^ CFU/ml). The canker lesions were evaluated 14 days after inoculation (DAI) in Valencia and Hamlin sweet orange leaves. Circles display the details of canker lesions observed under a GFP filter at the inoculation wound region (IWR). Bacterial populations (CFU/ml) evaluated with samples from IWR **(B)** and OWR **(C)** in *CsSAMT* lines and their respective WT at 7 and 14 DAI. At least three biological independent experiments with four internal replicates were performed. Asterisks (^*^) indicate significant differences based on Student’s *t*-test (*p* < 0.05) comparing transgenic lines and their respective wild type at each time point of IWR and OWR. VWT and HWT refer to Valencia and Hamlin WT cultivars, respectively.

To verify if plants overexpressing *CsSAMT* could promote resistance to *X. citri*, canker symptoms and the bacterial population were evaluated at 7 and 14 days after inoculation (DAI) of a green fluorescent protein (GFP)-expressing strain of *X. citri*. Although no difference in symptoms was observed at 7 DAI (Supplementary Figure 3), all *CsSAMT* lines showed reduced canker symptoms at 14 DAI compared to the WT (Figure 4A). Interestingly, the canker lesions in *CsSAMT* lines were restricted to the inoculation wound region (IWR), while WT developed typical canker symptoms, such as water soaking and erupted pustule lesions, over the infiltrated leaf area in both Valencia and Hamlin cultivars (Figure 4A). In addition, for Hamlin WT, which is a more susceptible variety (de Carvalho et al., 2015), more severe symptoms, such as abscission of the petiole, were observed, a characteristic not verified in HSAMT transgenic leaves (Figure 4A). Fluorescence microscopy analysis of GFP-expressing *X. citri* in the leaves of WT confirmed pathogen colonization throughout the infiltrated area, while in *CsSAMT* lines, it was restricted to the IWR (Figure 4A).

Since we observed the prevalence of canker formation at IWR in all *CsSAMT* lines, bacterial growth was evaluated by collecting leaf disk samples from IWR and out of the inoculation wound region (OWR; Supplementary Figure 3). For IWR, the bacterial population was significantly lower at 7 DAI in all *CsSAMT* lines, and at 14 DAI, a significant difference was observed only for HSAMT lines. However, the bacterial population present in the transgenic lines in OWR was significantly lower for all transgenic lines at 7 and 14 DAI (Figures 4B,C). Moreover, samples collected from OWR showed a prominent decrease in bacterial growth in all *CsSAMT* lines, reaching more than two logs compared to the WT (Figure 4C). These results indicate that overexpression of *CsSAMT* was able to restrict the bacteria to IWR in citrus, consequently decreasing canker symptom development compared to the WT.

### *CsSAMT* Overexpression Increases Resistance to *X. citri*

**Figure 5:**
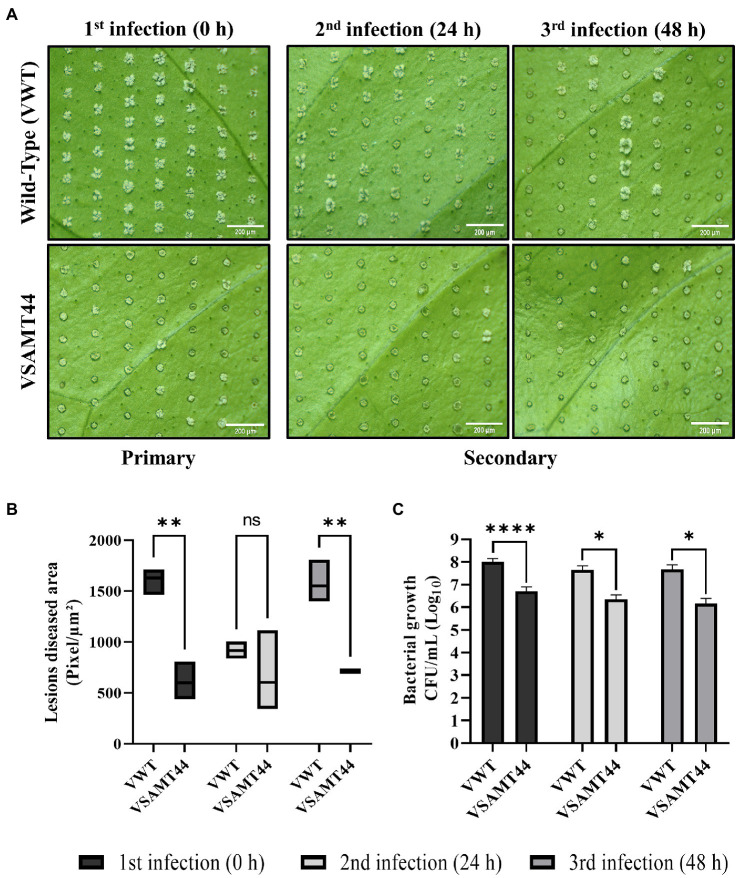
Canker lesion development and bacterial growth on leaves after *X. citri* primary and secondary infections. The pin-roller strategy was used for inoculation of 10^8^ cells/ml *X. citri. S*ymptoms were evaluated at 7 DAI for each *X. citri* inoculation time (0, 24, and 48 h). **(A)** Canker pustule formation in VWT and VSAMT44. **(B)** Statistical analysis of four representative diseased areas with 100 microinjuries per leaf and at least three leaves per treatment. **(C)** Growth of *X. citri* (CFU/ml) in primary and secondary infection sites on VWT and VSAMT44 leaves. Three biological independent experiments were performed. Asterisks indicate statistically significant differences based on Student’s *t*-test (^*^p < 0.05; ^**^p < 0.01; and ^****^p < 0.0001). Scale bar = 200 μm.

Since *CsSAMT* lines volatilized high amounts of MeSA, which is described as the mobile signal involved in SAR activation (Park et al., 2007), we evaluated if it could induce a defense priming response in citrus overexpressing *CsSAMT* (Supplementary Figure 4). Thus, one representative line, VSAMT44, was chosen for the analyses. Primary inoculation of *X. citri* was carried out on a leaf surface (0 h), and then, after 24 and 48 h, secondary inoculations were performed on other leaves of the same plants (Figure 1). For primary and secondary inoculations, VSAMT44 showed a significant reduction in canker symptoms (Figure 5). The canker lesions in VSAMT44 were milder than the canker lesions in WT and exhibited large white spongy pustules (Figure 5A). The VSAMT44 symptoms from the second and third infected leaves were mild and similar to those observed in the primary infection (Figure 5A). However, the WT decreased symptoms in the 24 h secondary infection, probably due to the defense activation from the primary infection. However, at 48 h, secondary infection of the WT plants showed large spongy pustules, similar to the primary infected leaf (Figures 5A,B).

Interestingly, VSAMT44 showed a reduction in pustule development at the primary and secondary (24 and 48 h) infections compared to the WT (Figures 5A,B), but this reduction was not significant at 24 h (Figure 5B). Moreover, the *X. citri* bacterial population was significantly lower in primary and secondary inoculation sites in VSAMT44 compared to WT (Figure 5C), which is in agreement with the infiltration experiments (Figure 4). The decrease in symptom development, together with the reduction in the bacterial population in all infection sites, suggests that defense response is already activated in VSAMT44, conferring a priming effect to the transgenic line, even before pathogen infection. Indeed, this is supported when comparing the primary and secondary infections within the WT and transgenic lines (Supplementary Figure 7).

### Transcriptional Regulation of Genes Involved in Salicylic Acid-Mediated Defenses Responses in the *CsSAMT* Line

**Figure 6:**
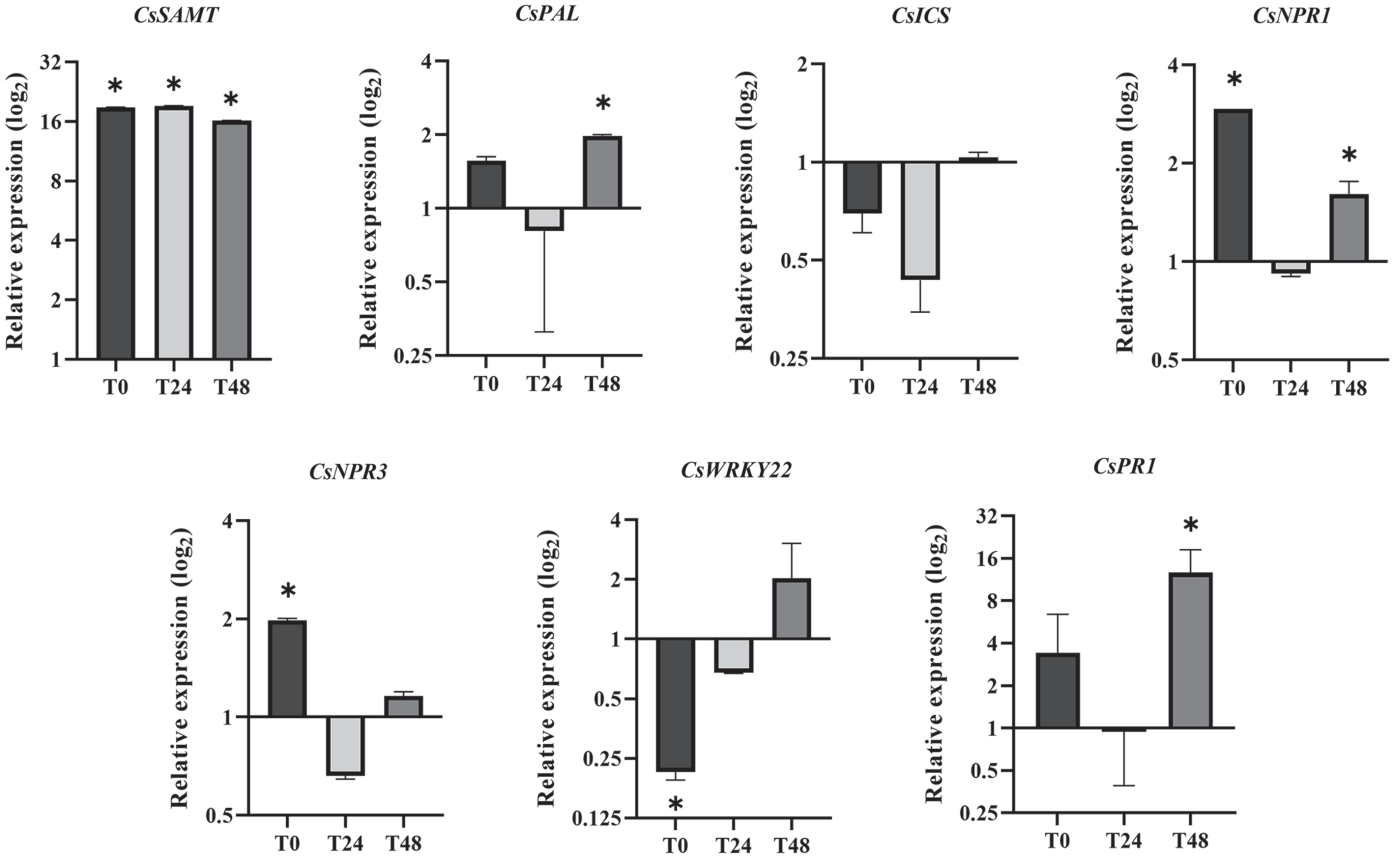
Expression of defense-associated genes in VSAMT44 in relation to VWT at T0, T24, and T48 (see Figure 1). Three biological independent experiments were performed, and the statistical significance of the means was calculated according to Student’s *t*-test (*p* < 0.05).

To expand the knowledge about the systemic activation of defense-related genes mediated by SAMT in citrus, qRT-PCR analysis was performed comparing VSAMT44 and VWT gene expression in noninfected leaves collected immediately before the primary (PI) and secondary (SI) inoculations (24 and 48 h after PI), named T0, T24, and T48, respectively (Figure 1). As expected, the expression of *CsSAMT* in VSAMT44 was significantly higher in all the leaves (Figure 6). The *PAL*-encoding gene, which is involved in SA biosynthesis and the phenylpropanoid pathway, showed higher expression in VSAMT44 at T48 compared to VWT. Conversely, *ICS*, another precursor of SA biosynthesis, was not modulated in the evaluated leaves, indicating that the PAL pathway is preferentially required in VSAMT44. In addition, *NPR1*, a key gene associated with SA-mediated defense responses, showed a significant higher expression in VSAMT44 T0, reinforcing the priming hypothesis. At T24, no modulation was observed for *NPR1*, which indicates that the presence of the pathogen modulates the expression of *NPR1* in VWT. However, *NPR1* expression was once again higher at T48 in VSAMT44 cells, and at that time point, *PR1* also showed higher expression than VWT (Figure 6). Moreover, expression of *NPR3* was higher only in VSAMT44 at T0, showing that the presence of free NPR1 monomers requires a high amount of NPR3, which is not observed in the presence of the pathogen (T24 and T48). Curiously, *CsWRKY22*, a transcription factor associated with susceptibility to citrus canker and SA (Kloth et al., 2016; Wang et al., 2019; Long et al., 2021), showed a significant lower expression in VSAMT44 at T0, when the pathogen was not present in the plant, suggesting that salicylic acid can modulate the expression of *CsWRKY22* and consequently reflect the reduced symptoms observed in the primary infection (Figure 6).

## Discussion

We have previously verified that overexpression of *CrSAMT* in tobacco increases MeSA production and leads to reduced symptom development of the citrus bacterial pathogen *X. fastidiosa* (Gómez et al., 2020). Due to the role of salicylic acid in plant defense responses to many bacterial diseases, the overexpression of *SAMT* could also confer resistance to different phytopathogens, such as *X. citri*, that cause citrus canker disease. Therefore, we generated Valencia and Hamlin sweet orange overexpressing *CsSAMT* to verify its potential to confer resistance to citrus canker, a very important citrus disease worldwide (Martins et al., 2020; Behlau, 2021). *CsSAMT* transgenic lines presented increased MeSA production for both cultivars. More recently, another transgenic variety of sweet orange, Wanjincheng, overexpressing *CsSAMT* also enhanced MeSA and SA production (Zou et al., 2021). These results prove that *CsSAMT* is functional and that its overexpression does not impair the normal development of at least three different *C. sinensis* cultivars. These results are in agreement with other transgenic plants overexpressing *SAMT*, such as tobacco, tomato, and soybean (Tieman et al., 2010; Lin et al., 2013, 2016; Gómez et al., 2020).

Transgenic plants showed enhanced resistance to *X. citri*, as observed by decreased canker symptoms and lower bacterial titers (Figure 4). In addition, the overexpression of *CsSAMT* in *C. sinensis* also enhanced tolerance to Huanglongbing, another important citrus bacterial disease (Zou et al., 2021). These resistance phenotypes could result from increasing MeSA production, which could be involved in SAR activation. Indeed, exogenous application of SA and SAR inducers has been shown to confer resistance to *X. citri* (Graham and Myers, 2011; Wang and Liu, 2012). Thus, to investigate the possible relationship between citrus canker resistance and systemic resistance, primary and secondary inoculations were used to verify symptom development. Furthermore, the expression of defense-related genes was determined using leaves collected immediately before each inoculation (T0, T24, and T48). As shown above (Figure 5; Supplementary Figure 7), no significant difference in symptoms was observed in the transgenic plants between the first, second, and third infections indicating that basal immunity was already activated in the transgenic line. In the transgenic plants, *CsSAMT* expression is driven by the FMV promoter and therefore, the protein is constitutively produced all over the plant. Therefore, differently from the classical priming mediated by SAR, signal translocation is not an issue in these plants since the increased production of MeSA all over could activate a widespread defense response.

As expected, in the primary infection site, VSAMT44 presented increased resistance compared to VWT, as verified by bacterial population and canker symptom development. Gene expression analysis in T0 showed significantly higher expression of *CsSAMT*, *CsNPR1*, and *CsNPR3* and repression of *CsWRKY22* in the transgenic line. *NPR1* plays a positive role in plant defense (Backer et al., 2019), and overexpression of *AtNPR1* from *Arabidopsis* in *C. sinensis* was demonstrated to increase the resistance to *X. citri* (Zhang et al., 2010; Boscariol-Camargo et al., 2016). However, overexpression of *NPR1* is detrimental to plant development (Silva et al., 2018). Thus, as NPR3 acts as a negative regulator of NPR1 (Moreau et al., 2012), its overexpression in VSAMT44 could explain the normal phenotype observed in the transgenic lines, possibly due to the regulation of *CsNPR1* levels by *CsNPR3*. Repression of *CsWRKY22* is directly associated with citrus canker resistance (Wang et al., 2019; Long et al., 2021), and overexpression of *CsWRKY22* in *Arabidopsis* mediated susceptibility to aphids, which is associated with the suppression of SA signaling (Kloth et al., 2016). These two studies point to the involvement of SA levels and *CsWRKY22* expression. In fact, these results corroborate our observation that plants overexpressing *CsSAMT* somehow repress *CsWRKY22* and are more resistant to citrus canker disease (Wang et al., 2019; Long et al., 2021). Curiously, the presence of the pathogen (T24) led to a decrease in expression of *CsPAL*, *CsNPR1*, and *CsPR1* defense-related genes in all the plants (Supplementary Figure 8), but repression was transient in the transgenic plants. As *X. citri* secretes effectors (Abe and Benedetti, 2016; Martins et al., 2020), we hypothesize that they lead to effector-triggered susceptibility in VWT, impairing defense responses. However, in VSAMT44, the steady state expression levels of *CsPAL*, *CsNPR1*, and *CsPR1* are higher (Supplementary Figure 8), indicating that overexpression of *CsSAMT* results in a priming effect mimicking SAR activation, and ultimately leading to a higher resistance phenotype. In addition, induction of *CsPAL* could be also related to production of secondary metabolites involved in plant defense responses (Yadav et al., 2020). In summary, the overexpression of *CsSAMT* increased MeSA production and induced immune responses in transgenic plants at both primary and secondary infection sites. In SAR, the mobile signal is translocated from the primary infection site to distal parts of the plant. In the transgenic plants, the overexpression of SAMT might mimic the SAR response since it leads to a widely distributed production of MeSA and consequently an increased pathogen resistance.

This study reinforces the key role of SAMT in increasing resistance against phytopathogens in different crops (Tieman et al., 2010; Lin et al., 2013, 2016; Gómez et al., 2020). For citrus, this is a powerful tool since it has been demonstrated resistance against three bacterial pathogens that affect the industry of utmost importance (Gabriel et al., 2020; Gómez et al., 2020; Zou et al., 2021; this study). This resistance mediated by MeSA signaling prompts the plant to respond more efficiently to pathogen attacks without compromising plant development. In addition, MeSA-overproducing plants may have the potential to distribute a long-distance airborne signal able to induce SAR in neighboring plants (Shulaev et al., 1997; Heil and Ton, 2008; Yoneya and Takabayashi, 2014; Khait et al., 2018; Wenig et al., 2019; Masimbula et al., 2020), which needs to be further investigated for citrus.

## Conclusion

Salicylic acid has a crucial role in plant defense and therefore, modulating its level in plants could lead to enhanced resistance against phytopathogens. In the present study, we evaluated sweet orange cultivars (Valencia and Hamlin) overexpressing *CsSAMT*, leading to production of volatile MeSA without compromising plant development. This could lead to increase in the levels of salicylic acid and represents a possible strategy for controlling citrus diseases such as citrus canker. In fact, plants overexpressing *CsSAMT* show enhanced resistance to *X. citri* in both primary and secondary infection sites. In addition, we observed an expression response of defense-related genes differing from the WT plants, indicating that overexpression of *CsSAMT* could lead to a priming state. This is a promising approach to have higher resistance to citrus canker in the field in special because MeSA is a volatile molecule that can induce defense responses in neighbor plants as already reported for other plant species.

## Data Availability Statement

The original contributions presented in the study are included in the article/Supplementary Materials, further inquiries can be directed to the corresponding author.

## Author Contributions

AS conceived and designed the research. AS, MM, and MT provided reagents and analytical tools and discussed the data. CN and RC performed the experiments. CN and NT-S analyzed data. CN, NT-S, and AS wrote the manuscript. All authors contributed to the article and approved the submitted version.

## Funding

This work was supported by a research grant from the Fundação de Amparo à Pesquisa do Estado de São Paulo (FAPESP—2013/10957-0). CN, a Ph.D. student from the Graduate Program in Genetics and Molecular biology (UNICAMP), was supported by a fellowship from (CAPES-Grant 001). NT-S and RC are postdoctoral fellows supported by FAPESP (2019/01447-5 and 2017/16142-0). MT, MM, and AS are recipients of research fellowships from Conselho Nacional de Desenvolvimento Científico e Tecnológico (CNPq).

## Conflict of Interest

The authors declare that the research was conducted in the absence of any commercial or financial relationships that could be construed as a potential conflict of interest.

## Publisher’s Note

All claims expressed in this article are solely those of the authors and do not necessarily represent those of their affiliated organizations, or those of the publisher, the editors and the reviewers. Any product that may be evaluated in this article, or claim that may be made by its manufacturer, is not guaranteed or endorsed by the publisher.
