# Supplementary material for: Overexpression of CsSAMT in Citrus sinensis Induces Defense Response and Increases Resistance to Xanthomonas citri subsp. citri
Source: Front Plant Sci. 2022 Mar 24;13:836582. doi: 10.3389/fpls.2022.836582 (PMC8988300; doi:10.3389/fpls.2022.836582)
Supplement: Supplementary file 1 [file Data_Sheet_1.PDF]

Supplementary Material

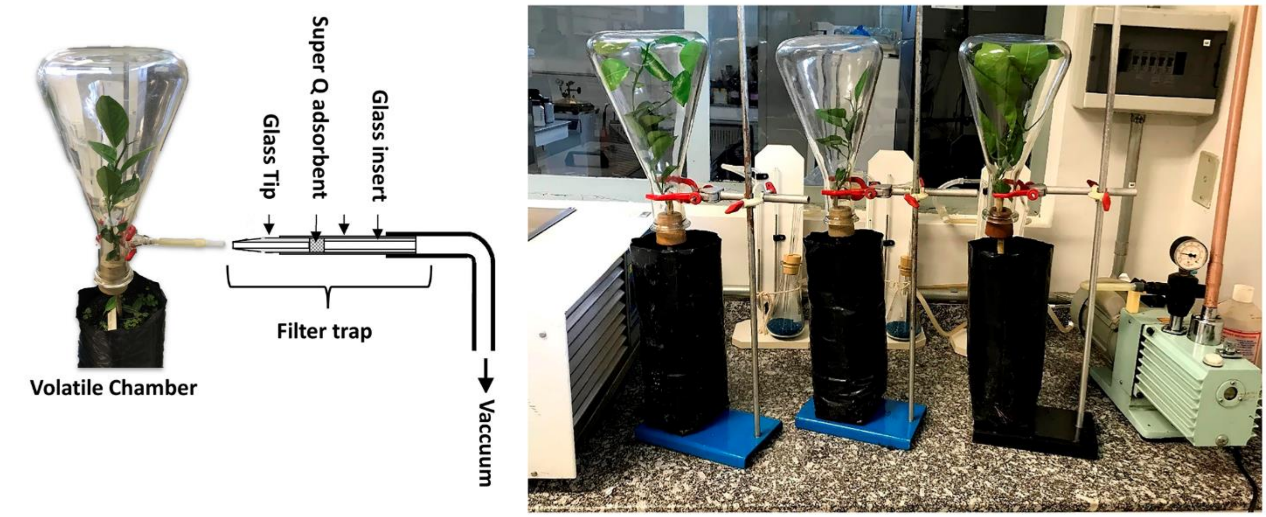

SUPPLEMENTARY FIGURE 1. Volatile collection system. Three biological replicates were placed into parallel chamber flask glasses. The leaf volatiles were forced to a Super Q trap by a controlled vacuum continuously for 24 hours.

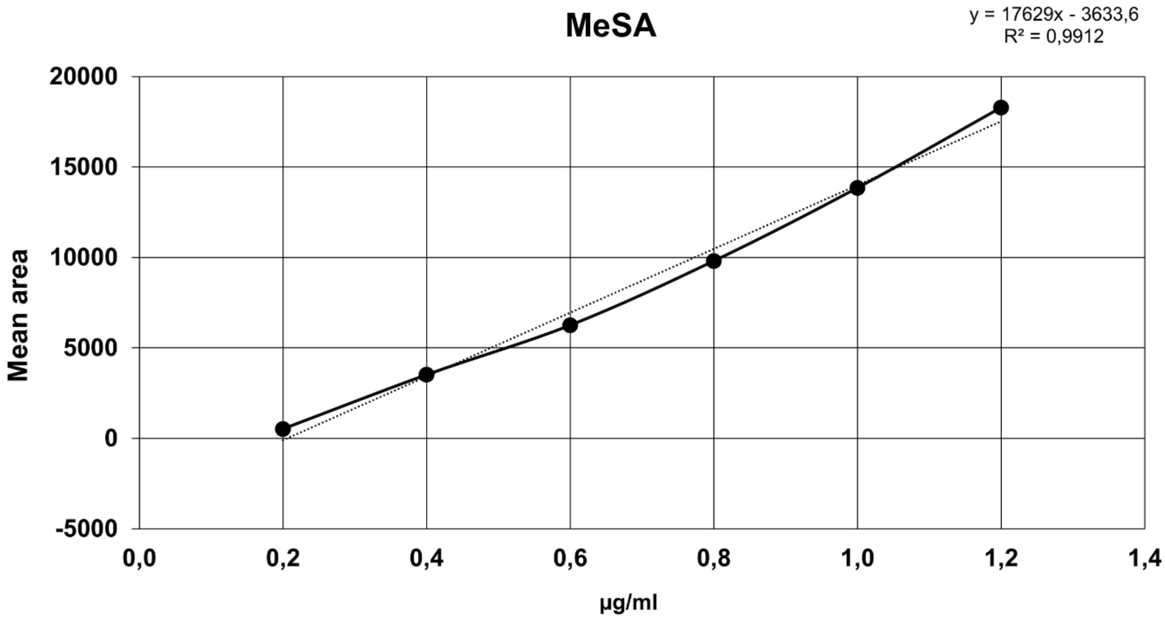

SUPPLEMENTARY FIGURE 2. MeSA calibration curve using dilutions of known amounts of standard MeSA diluted in dichloromethane at ranges varying from 1.2 µg/mL to 0.2 µg/mL with R^2 of 0.9912.

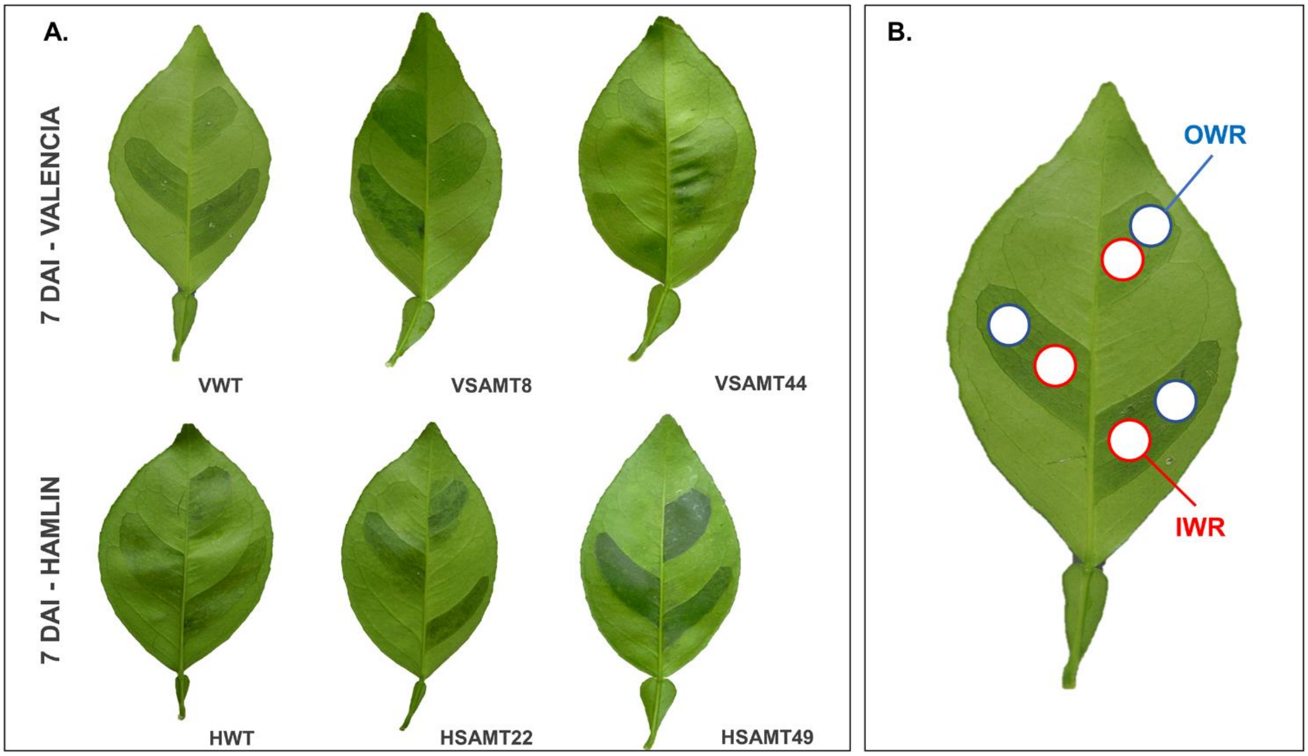

SUPPLEMENTARY FIGURE 3. Canker symptoms in CsSAMT lines and WT plants evaluated 7 days after inoculation (DAI). **(A)** Fully expanded leaves of CsSAMT lines and the wild types (VWT and HWT) were infiltrated with *X. citri* 306_GPF (10^4 CFU/mL). **(B)** Representative detached leaf demonstrating where samples were excised at the inoculation wound region (IWR) (red halo circles) and out of the inoculation wound region (OWR) (blue halo circles) within each inoculated area.

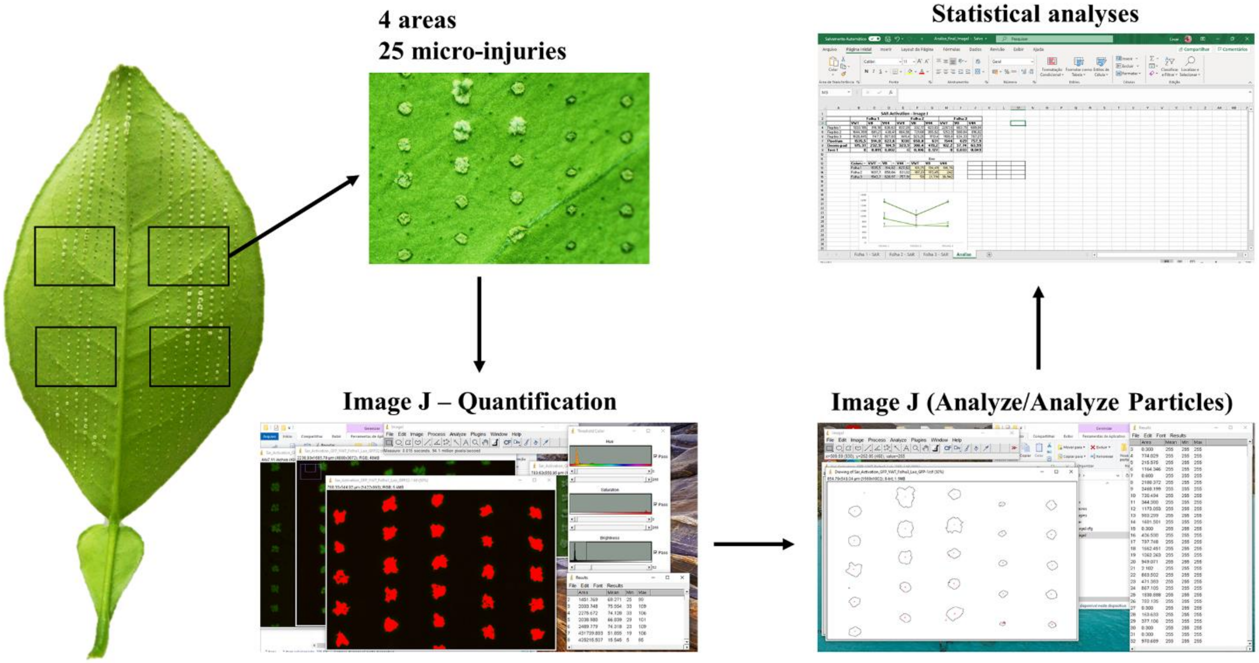

SUPPLEMENTARY FIGURE 4. Representative quantification of canker symptoms evaluated 7 days after the primary and secondary inoculations. At least three biological experiments with four internal replicates were analyzed for canker lesions by measuring the pixels using ImageJ software (Analyze/Analyze particles). Statistical analyses were performed using two-way ANOVA with Tukey's test ($P < 0.05$) in GraphPad Prism 9 for Windows.

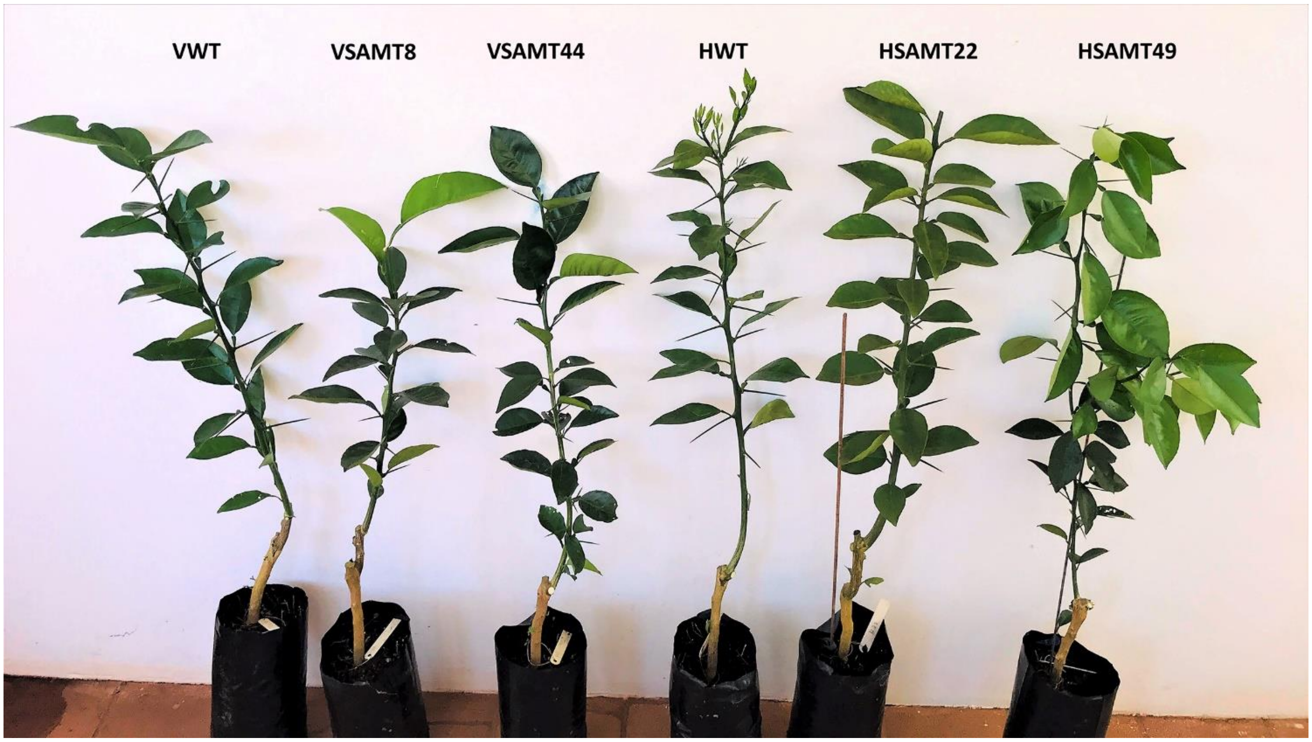

SUPPLEMENTARY FIGURE 5. Phenotypes of regenerated plants. The transgenic and wild-type plants were regenerated *in vitro* and grafted into Rangpur lime rootstocks. The plants were maintained in a greenhouse for one year and exhibited normal phenotypes with no obvious abnormalities.

A.

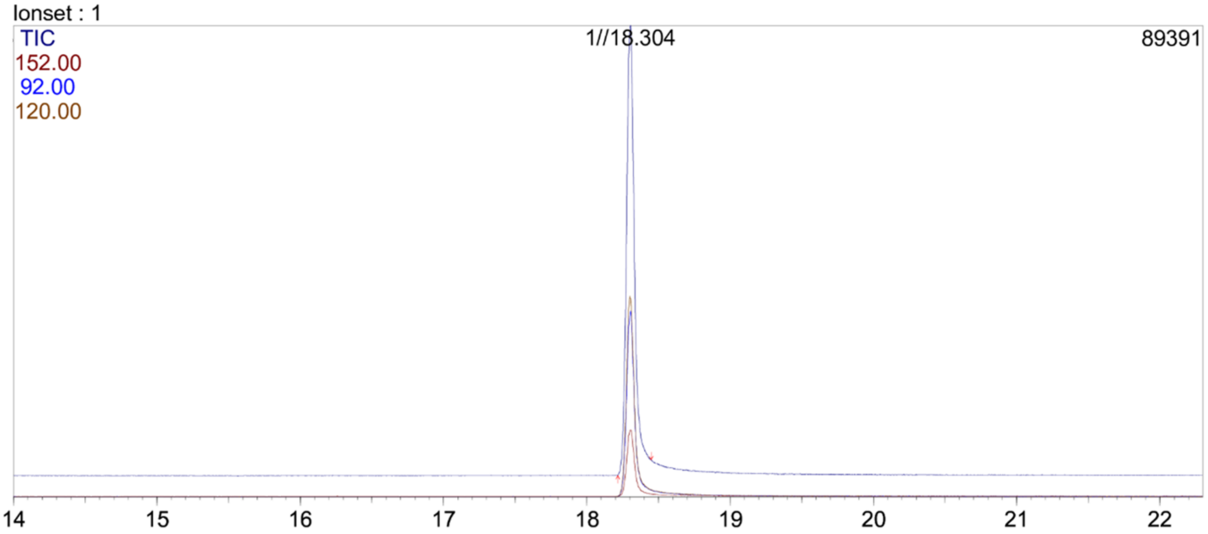

B.

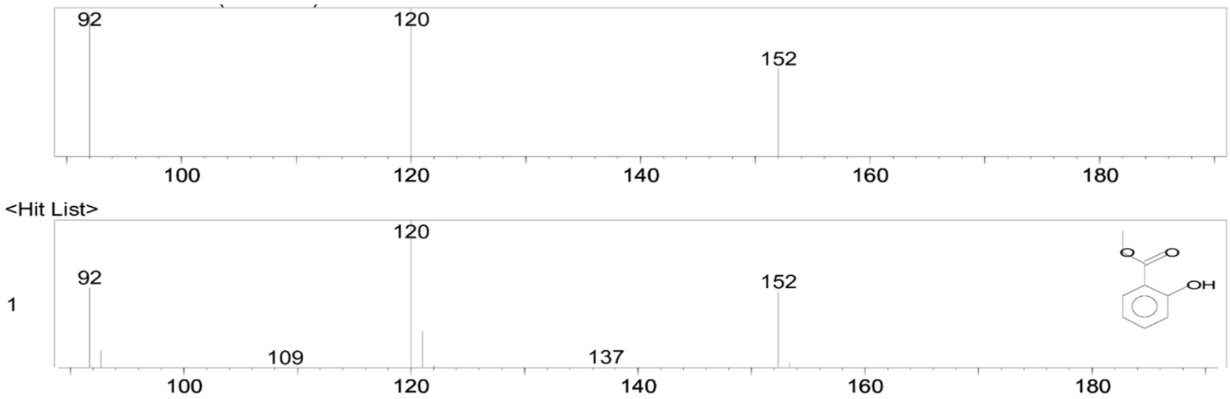

C.

No	SI	Mol.Wgt.	Mol.Form./Compound Name	CAS No.	Entry	LIB#
1	86	152	$C_8H_8O_3$ Methyl Salicylate	119-36-8	4663	1
2	86	194	$C_{10}H_{10}O_4$ Benzoic acid, 2-(acetyloxy)-, methyl ester (CAS) ACETYL METHYL SALICYLATE $$ O-Acetyl	580-02-9	44538	3
3	85	152	$C_8H_8O_3$ Methyl Salicylate	119-36-8	4664	1
4	85	152	$C_8H_8O_3$ Benzoic acid, 2-hydroxy-, methyl ester, ion(1-) (CAS) Benzoic acid, 2-hydroxy-, methy	119-36-8	22301	3

SUPPLEMENTARY FIGURE 6. Chromatogram of standard MeSA; **(A)** retention time at 18.304 min. **(B)** Mass spectra of MeSA selected ion monitoring (*m/z* 92, *m/z* 120 and m*/z* 150). **(C)** GC/MS system database NIST 62 Library.

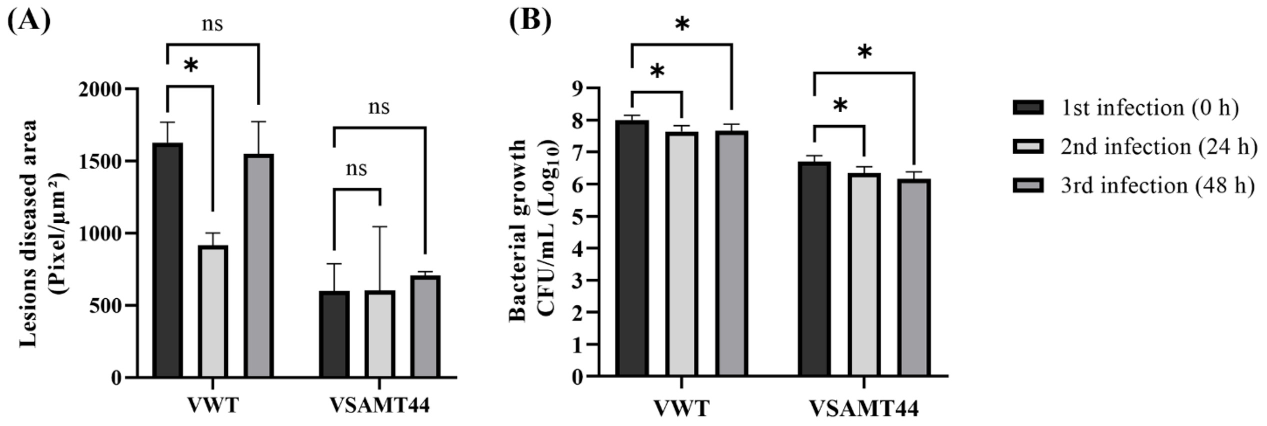

SUPPLEMENTARY FIGURE 7. Symptoms and *X. citri* bacterial population evaluation in leaves of VWT and transgenic lines after primary (0) and secondary infections (24 and 48h). **(A)** Statistical analysis of four representative diseased areas with 100 microinjuries per leaf and at least three leaves per treatment. **(B)** Growth of *X. citri* (CFU/mL) in primary and secondary infection sites on VWT and VSAMT44 leaves. Three biological independent experiments were performed. Asterisks indicate statistically significant differences based on Student's *t*-test (* $P < 0.05$). This figure represents another analysis of FIGURE 5 in the main manuscript.

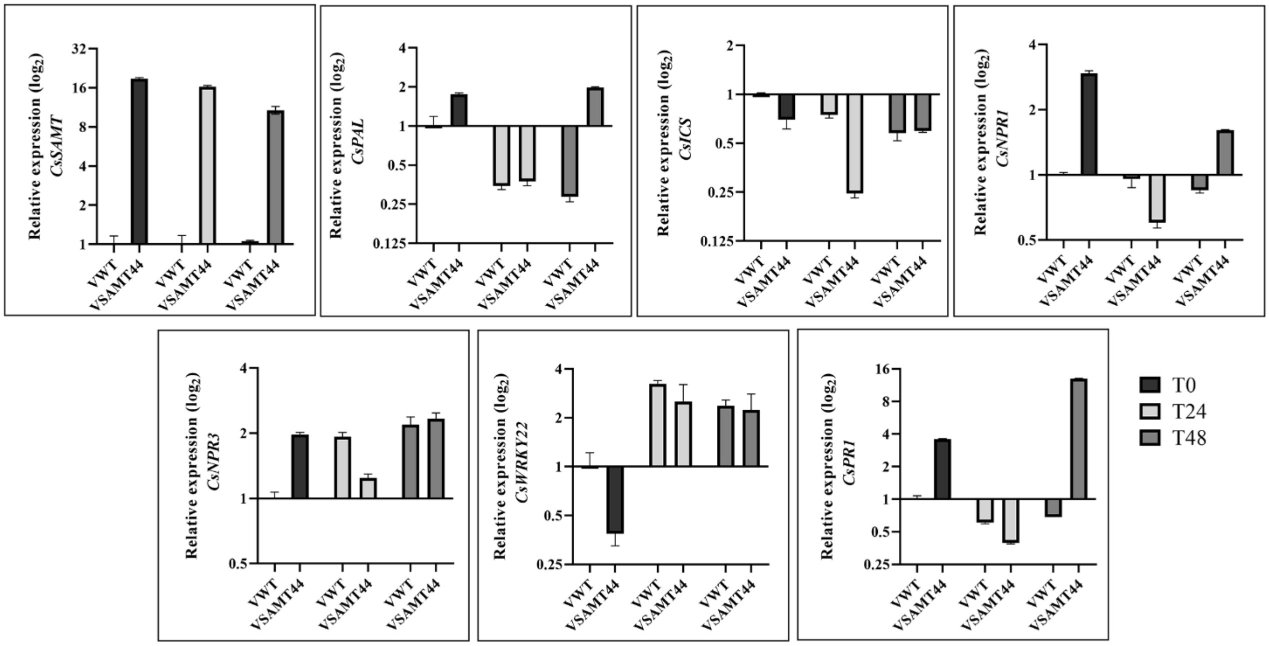

SUPPLEMENTARY FIGURE 8. Expression analysis of SA-associated genes in VSAMT44 and VWT plants in relation to T0 of VWT. This figure represents another analysis of FIGURE 6 in the main manuscript.
